# Head and neck cancer incidence is rising but the sociodemographic profile is unchanging: a population epidemiological study (2001–2020)

**DOI:** 10.1038/s44276-024-00089-z

**Published:** 2024-09-17

**Authors:** Craig D. L. Smith, Alex D. McMahon, Mitana Purkayastha, Grant Creaney, Kelten Clements, Gareth J. Inman, Lesley A. Bhatti, Catriona M. Douglas, Claire Paterson, David I. Conway

**Affiliations:** 1 School of Medicine, Dentistry, and Nursing, University of Glasgow, Glasgow, UK; 2 School of Cancer Sciences, University of Glasgow, Glasgow, UK; 3 Glasgow Head and Neck Cancer (GLAHNC) Research Group, Glasgow, UK; 4 Great Ormond Street Institute of Child Health, University College London, London, UK; 5 Cancer Research UK Scotland Institute, Glasgow, UK; 6 Public Health Scotland, Edinburgh, UK; 7 Department of Otolaryngology/Head and Neck Surgery – Glasgow Royal infirmary and Queen Elizabeth University Hospital, Glasgow, UK; 8 Beatson West of Scotland Cancer Centre, Glasgow, UK

## Abstract

**Background:**

Increasing incidence of head and neck cancers (HNCs), driven by rising rates of oropharynx cancer (OPC), has been recorded around the world. This study examined trends in HNC and subsites (oral cavity, oropharynx, and larynx cancers) in Scotland focusing on assessing whether the sociodemographic profile has changed over the past 20 years.

**Methods:**

Scottish Cancer Registry data (2001–2020) including European Age Standardised Rates of HNC and subsites were analysed in multivariate Poisson regression by age, sex, area-based socioeconomic status, and year of diagnosis (with interaction tests).

**Results:**

Overall HNC and oral cavity cancer (OCC) incidence remained relatively stable. OPC incidence rates increased by 78%, while larynx cancer incidence declined by 27%. Over time, there were marginal shifts to a slightly older age profile for HNC (*p* = 0.001) and OCC (*p* = 0.001), but no changes in OPC (*p* = 0.86) and larynx cancer (*p* = 0.29). No shift in the sex profile of HNC was observed except for minor increases in female OCC rates (*p* = 0.001), and the socioeconomic distribution remained unchanged across all HNC subsites.

**Conclusions:**

There have been no significant changes in the sociodemographic profile of HNC in Scotland over the last 20 years, despite the changing trends in HNCs with dramatically increasing incidence rates in OPC and reducing larynx cancer. This information can be used to target or stratify HNC prevention and control.

## Introduction

Squamous cell carcinomas of oral cavity, larynx and pharynx are commonly known as head and neck cancers (HNC). As the seventh most common cancer globally, it is a growing and increasing public health challenge with over 800,000 incident cases and 400,000 deaths in 2020 [1–3].

In the UK patterns are in keeping with this; since the early 1990s HNC incidence has risen by 37%, with approximately 12,400 cases and 4100 deaths annually [4, 5]. Incidence rates of HNC vary by geographical region and HNC subsite; large increases have been observed in oropharyngeal cancer rates in the UK as a whole (ranging from 45% to an 85% increase in Scotland). This has been accompanied by reports of modest increases in oral cavity cancer rates and declines in larynx cancer rates [6–8]. Wide socioeconomic inequalities in HNC incidence are observed, with the highest rates consistently found among those most socioeconomically deprived groups [6]. HNC is more common among older people and men are typically at a two-four-fold increased risk of head and neck cancer compared with women [1, 9].

In recent years, the increase in oropharyngeal cancer rates has become a global phenomenon, and largely attributed to Human Papilloma Virus (HPV), particularly in the United States and Europe [4, 6, 7, 10]. Within these trends, there are emerging reports of changes in the sociodemographic profile of people with head and neck cancer, especially oropharyngeal cancer. Some of these studies suggest the demographics of patients with OPC are younger, female, with higher socioeconomic status (SES) and presenting without a history of smoking or alcohol consumption, the traditional risk factors for HNC [11–19].

Here, we aimed to investigate the trends in incidence and sociodemographic profile (age, sex, and area-based socioeconomic deprivation) of cancers of the oral cavity, oropharynx, and larynx over time at a population-level.

## Methods

Scottish Cancer Registry data for the years 2000–2021 were accessed including: Incident cases of HNC and its subsites – defined using international classification of disease (tenth edition ICD-10) codes and grouped as: oral cavity (inner lip – C00.3–C00.9, dorsal, overlapping or NOS tongue – C02, gingivae – C03, floor of mouth – C04, soft palate, uvula, palate or NOS – C05 and cheek, other and NOS mouth – C06); oropharynx (base of tongue – C01, lingual tonsil – C02.4, tonsil – C09, oropharynx – C10, pharynx – C14.0,14.2); larynx (C32); and all HNC was defined as the above sites in addition to tumours of the nasopharynx (C11), piriform sinus (C12), Hypopharynx (C13) and other overlapping sites (C14.8) [20–22].

Available sociodemographic factors of cases were also included: age (in five-year bands), sex (male / female), and an area-based index of multiple deprivation – the Scottish Index of Multiple Deprivation (SIMD) [23]. This small area-based deprivation index is calculated from several domains including income, employment, education, health, access to services, crime, and housing. In the Scottish Cancer Registry, this decile-based data were divided into fifths, where SIMD-1 was the most socioeconomically deprived and SIMD-5 was the least socioeconomically deprived [24].

European age-standardised rates were calculated for all subsites, age, sex, SIMD, and year of diagnosis. Using Poisson regression, rate ratios were calculated to describe and compare HNC incidence by subsite, age, sex, SIMD, and year of diagnosis. Age-standardised incidence rates were plotted by subsite to visualise trends in HNC incidence. In order to assess for temporal changes in incidence, interaction tests were also conducted for the 5-year aggregated data. All statistical analyses were conducted with SAS version 9.4.

## Results

In Scotland there were 20,850 HNC cases identified by the Scottish Cancer Registry from 2001 to 2020. Of these cases, 70.5% (*n* = 14,706) were male and 29.5% (*n* = 6,144) were female. The crude number of cases and age standardised incidence rate per 100,000 for each subsite is summarised in Table 1 by age, sex, SIMD, region and year of diagnosis. Substantial increases in age standardised oropharynx cancer (OPC) incidence and declines in larynx cancer rates were observed. The age standardised incidence rate of HNC was greatest among 60–64-year-olds (3.41 cases per 100,000). OPC incidence peaked in 60–64-year-olds (1.00 per 100,000), whilst OCC incidence (1.17 per 100,000) and larynx cancer incidence (1.07 per 100,000) peaked among 65–69-year-olds.

**Table 1:** Counts and Age-Standardised Incidence Rates for HNC and subsites by year, age, sex, SIMD (with peak incidence in bold).

	HNC		OPC		OCC		Larynx	
	N	EASR	N	EASR	N	EASR	N	EASR
**Year**								
2001	885	20.09	160	3.54	318	7.25	**318**	**7.27**
2002	881	19.81	162	3.58	327	7.37	291	6.59
2003	903	20.04	167	3.64	352	7.77	294	6.63
2004	933	20.57	192	4.20	335	7.39	310	6.90
2005	893	19.40	191	4.03	340	7.45	283	6.24
2006	968	20.84	207	4.34	336	7.27	321	6.98
2007	987	20.92	214	4.44	366	7.80	300	6.39
2008	937	19.56	216	4.42	357	7.50	281	5.92
2009	1058	21.74	288	5.71	371	7.72	288	6.02
2010	1044	21.23	258	5.11	379	7.76	309	6.40
2011	1062	21.35	275	5.41	368	7.42	296	6.06
2012	**1164**	**22.94**	341	6.61	401	7.95	283	5.64
2013	1139	22.31	330	6.30	412	8.14	289	5.75
2014	1111	21.48	304	5.75	**423**	**8.22**	297	5.81
2015	1164	22.17	339	6.32	409	7.85	297	5.70
2016	1112	21.02	318	5.92	400	7.63	285	5.39
2017	1153	21.40	369	6.76	397	7.42	272	5.08
2018	1217	22.39	399	7.23	407	7.55	288	5.32
2019	1126	20.45	390	7.03	342	6.25	276	5.04
2020	1113	20.07	**401**	**7.17**	368	6.65	228	4.14
**Age**								
Under 25	64	0.06	6	0.01	33	0.03	3	0.00
25–29	48	0.04	7	0.01	27	0.02	6	0.01
30–34	80	0.08	18	0.02	39	0.04	11	0.01
35–39	222	0.22	66	0.06	84	0.08	41	0.04
40–44	502	0.47	166	0.16	198	0.19	93	0.09
45–49	1095	1.00	417	0.38	370	0.34	215	0.20
50–54	2049	1.93	811	0.77	604	0.57	433	0.41
55–59	2990	2.81	1016	0.95	926	0.87	757	0.71
60–64	**3507**	**3.41**	**1030**	**1.00**	1149	1.12	984	0.96
65–69	3333	3.36	775	0.78	**1158**	**1.17**	**1064**	**1.07**
70–74	2816	3.06	563	0.61	1019	1.11	919	1.00
75–79	2029	2.27	370	0.41	766	0.86	697	0.78
80–84+	2115	2.28	277	0.30	1035	1.12	582	0.63
**Sex**								
Male	**14706**	**32.03**	**4135**	**8.63**	**4377**	**9.63**	**4593**	**10.25**
Female	6144	11.47	1387	2.59	3031	5.64	1212	2.28
**SIMD**								
SIMD: 1	**6290**	**36.04**	**1557**	**8.76**	**2005**	**11.34**	**2023**	**11.88**
SIMD: 2	4867	25.49	1193	6.15	1682	8.65	1492	8.03
SIMD: 3	3972	19.80	1107	5.41	1457	7.15	1023	5.27
SIMD: 4	3275	16.39	992	4.78	1249	6.20	741	3.92
SIMD: 5	2446	12.92	673	3.36	1015	5.33	526	2.97

*HNC* All Head and Neck Cancer, *OPC* Oropharyngeal Cancer, *OCC* Oral Cavity Cancer, *Larynx* Larynx Cancer, *N* Number, *EASR per 100,000* European Age-Standardised Rate per 100,000 persons, *SIMD* Scottish Index of Multiple Deprivation, where SIMD-1 was the most socioeconomically deprived and SIMD-5 was the least socioeconomically deprived.

**Fig. 1:**
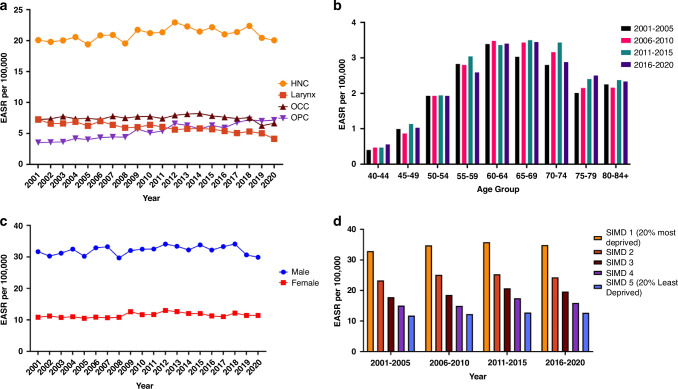
a–d Combined Plot of Age-Standardised HNC Incidence Trends (2001-2020). (**a**) Age-Standardised Overall HNC and Subsite Incidence Trends (2001-2020), (**b**) Age-standardised Incidence of HNC by Age-group (2001-2020), (**c**) Age-Standardised Incidence of HNC by Sex (2001-2020) and (**d**) Age-standardised Incidence of HNC by SIMD Quintile (2001-2020). HNC All Head and Neck Cancer, OPC Oropharyngeal Cancer, OCC Oral Cavity Cancer, Larynx Larynx Cancer, EASR per 100,000 European Age-Standardised Rate per 100,000 persons, SIMD Scottish Index of Multiple Deprivation, where SIMD-1 was the most socioeconomically deprived and SIMD-5 was the least socioeconomically deprived.

Table 2 details Poisson regression rate-ratios and interaction tests for each subsite by age, sex, SIMD, and year of diagnosis in 5-year periods. From 2001–2005 to 2016–2020, the overall age-standardised HNC incidence rate increased by 5%, (RR = 1.05, 95% CI = 1.01–1.09). (Fig. 1a) Within this same period, OPC incidence rates increased substantially by 78% (RR = 1.78, 95% CI = 1.65–1.93). Oral cavity cancer (OCC) incidence rates remained relatively stable, exhibiting a modest but non-statistically significant decrease during the overall study period by 23% (RR = 0.94, 95% CI = 0.88–1.01), while larynx cancer incidence rates decreased significantly by 27% (RR = 0.73, 95% CI = 0.68–0.79)

**Table 2:** Adjusted rate-ratios and interaction tests for HNC and subsite incidence by Age, Sex, SIMD and Year of Diagnosis (5-year period).

	HNC				OPC				OCC				Larynx			
	RR	95%CI Lower	95%CI Upper	*p*	RR	95% CI Lower	95% CI Upper	*p*	RR	95% CI Lower	95%CI Upper	*p*	RR	95% CI Lower	95%CI Upper	*p*
**Interaction**																
Age*Year	–	–	–	**0**.**001**	–	–	–	0.86	–	–	–	**0.001**	–	–	–	0.29
Sex*Year	–	–	–	0.55	–	–	–	0.22	–	–	–	**0.01**	–	–	–	0.59
SIMD*Year	–	–	–	0.75	–	–	–	0.55	–	–	–	0.23	–	–	–	0.15
**Year**																
2001–2005	REF	REF	REF		REF	REF	REF		REF	REF	REF		REF	REF	REF	
2006–2010	1.04	1.00	1.08	0.05	1.27	1.16	1.38	**<0.0001**	1.02	0.95	1.09	0.65	0.94	0.87	1.01	0.08
2011–2015	1.10	1.05	1.14	**<0.0001**	1.59	1.47	1.73	**<0.0001**	1.06	0.99	1.13	0.10	0.85	0.79	0.91	**<0.0001**
2016–2020	1.05	1.01	1.09	**0.03**	1.78	1.65	1.93	**<0.0001**	0.94	0.88	1.01	0.08	0.73	0.68	0.79	**<0.0001**
**Age**																
Under 25	0.01	0.01	0.02	**<0.0001**	0.00	0.00	0.01	**<0.0001**	0.02	0.02	0.03	**<0.0001**	0.00	0.00	0.01	**<0.0001**
25–29	0.05	0.03	0.06	**<0.0001**	0.02	0.01	0.04	**<0.0001**	0.08	0.05	0.12	**<0.0001**	0.03	0.01	0.06	**<0.0001**
30–34	0.08	0.06	0.10	**<0.0001**	0.05	0.03	0.07	**<0.0001**	0.11	0.08	0.16	**<0.0001**	0.05	0.03	0.10	**<0.0001**
35–39	0.21	0.18	0.25	**<0.0001**	0.17	0.13	0.22	**<0.0001**	0.24	0.19	0.30	**<0.0001**	0.20	0.14	0.27	**<0.0001**
40–44	0.47	0.42	0.52	**<0.0001**	0.41	0.34	0.49		0.54	0.46	0.65	**<0.0001**	0.43	0.34	0.55	**<0.0001**
45–49	REF	REF	REF		REF	REF	REF		REF	REF	REF		REF	REF	REF	
50–54	1.92	1.79	2.07	**<0.0001**	1.98	1.76	2.23	**<0.0001**	1.68	1.48	1.92	**<0.0001**	2.09	1.77	2.46	**<0.0001**
55–59	3.01	2.81	3.23	**<0.0001**	2.66	2.37	2.98	**<0.0001**	2.77	2.46	3.12	**<0.0001**	3.91	3.36	4.55	**<0.0001**
60–64	3.97	3.71	4.24	**<0.0001**	3.02	2.70	3.39	**<0.0001**	3.85	3.42	4.33	**<0.0001**	5.72	4.93	6.62	**<0.0001**
65–69	4.30	4.01	4.60	**<0.0001**	2.59	2.30	2.92	**<0.0001**	4.41	3.92	4.96	**<0.0001**	7.07	6.11	8.19	**<0.0001**
70–74	4.37	4.08	4.69	**<0.0001**	2.28	2.01	2.58	**<0.0001**	4.64	4.12	5.23	**<0.0001**	7.39	6.37	8.57	**<0.0001**
75–79	4.17	3.87	4.48	**<0.0001**	2.00	1.74	2.30	**<0.0001**	4.54	4.01	5.14	**<0.0001**	7.48	6.42	8.71	**<0.0001**
80–84+	3.65	3.39	3.92	**<0.0001**	1.25	1.08	1.46	**0.0035**	4.97	4.41	5.60	**<0.0001**	5.47	4.68	6.40	**<0.0001**
**Sex**																
Male	2.83	2.74	2.91	**<0.0001**	3.35	3.16	3.57	**<0.0001**	1.74	1.66	1.82	**<0.0001**	4.57	4.29	4.87	**<0.0001**
Female	REF	REF	REF		REF	REF	REF		REF	REF	REF		REF	REF	REF	
**SIMD**																
SIMD: 1 (20% most deprived)	2.87	2.73	3.00	**<0.0001**	2.60	2.38	2.85	**<0.0001**	2.18	2.02	2.35	**<0.0001**	4.28	3.89	4.71	**<0.0001**
SIMD: 2	2.02	1.92	2.12	**<0.0001**	1.84	1.67	2.02	**<0.0001**	1.66	1.53	1.79	**<0.0001**	2.85	2.58	3.15	**<0.0001**
SIMD: 3	1.56	1.48	1.64	**<0.0001**	1.60	1.45	1.76	**<0.0001**	1.37	1.27	1.49	**<0.0001**	1.85	1.67	2.06	**<0.0001**
SIMD: 4	1.28	1.22	1.35	**<0.0001**	1.42	1.28	1.56	**<0.0001**	1.18	1.09	1.28	**<0.0001**	1.34	1.20	1.50	**<0.0001**
SIMD: 5 (20% least deprived)	REF	REF	REF		REF	REF	REF		REF	REF	REF		REF	REF	REF	

*HNC* All Head and Neck Cancer, *OPC* Oropharyngeal Cancer, *OCC* Oral Cavity Cancer, *Larynx* Larynx Cancer, *RR* Rate Ratio (derived from Poisson Regression), *95% CI* 95% Confidence Interval, * interaction test, *REF* Reference Category, *SIMD* Scottish Index of Multiple Deprivation, where SIMD-1 was the most socioeconomically deprived and SIMD-5 was the least socioeconomically deprived.

Significant *p*-values highlighted in bold.

Overall, males were observed to have significantly higher HNC incidence rates (Fig. 1c) (RR = 2.83, 95% CI 2.74–2.91). This effect was also observed across all subsites (Table 2); male incidence rates were over three times that of females for OPC (RR = 3.35, 95% CI 3.16–3.57), over one and a half times greater for OCC (RR = 1.74, 95% CI 1.66–1.82) and over four times greater times for larynx cancer (RR = 4.57, 95% CI 4.30–4.87).

Temporally, HNC incidence rates by SIMD have remained stable. Large inequalities persist with the highest incidence rates of HNC observed among those most socioeconomically deprived (Fig. 1d). (RR = 2.87, 95% CI 2.74–2.91). The incidence rate trends by SIMD reflect the trends of each subsite (for example, increasing rates across all SIMD quintiles for OPC) but within each sub-site, the same strong inequality gradient remained true.

As can be seen from the Fig. 1a–d and Supplementary Figs. 1–9, there have been no major shifts in the peak incidence rates by age-groups, SIMD, or sex. Interaction tests conducted within the Poisson models were suggestive of no temporal associations in the relationship between sex and HNC incidence (*p* = 0.55). This remained true for cancers of the oropharynx (*p* = 0.22) and larynx (*p* = 0.59). A significant interaction was observed when assessing OCC incidence by sex over time, with small increases and declines in the burden of female and male cases, respectively (*p* = 0.01). There were no temporal changes or associations observed when assessing HNC incidence by SIMD over time (*p* = 0.75). This remained true for all subsites (OPC: *p* = 0.55, OCC: *p* = 0.23, Larynx: *p* = 0.15). Temporal associations of increasing cancer incidence with age over time varied by subsite. No temporal interactions were observed for OPC cases (*p* = 0.86) or larynx cancer by age-group over time (*p* = 0.29). The interactions tests were suggestive of fluctuations in OCC incidence by age over time (*p* = 0.001). However, upon interpretation of plots and table values, there was no consistent pattern change in OCC incidence, although there was a marginal shift to a slightly older peak age (65–69 years) (Supplementary Fig. 2). At an overall level, a significant temporal interaction was associated with age and HNC incidence (*p* = 0.001). This could be explained by a similar, but also marginal, shift to a slightly older peak-age group (65–69 years). A consistent increase in patients aged 75–79 years old was also observed over time (Fig. 1b).

## Discussion

This study shows that the predominant sociodemographic profile of people with all types of HNC is consistently males in their early to mid-60s (and older) from low socioeconomic backgrounds and this has remained unchanged over the last 20 years. Over this period, OPC incidence rates have risen while rates of larynx cancer have declined, and OCC rates have remained stable. In this changing makeup of head and neck cancer diagnoses and despite differing aetiologies, the underlying sociodemographic profile is unchanged.

Our results contrast with previous reports which have suggested a changing sociodemographic profile of HNC [11]. We found no evidence of changing sex distribution of HNCs overall, with incidence rates consistently greater among men for all subsites and across the study period – consistent with previous studies [25–27]. A significant interaction was observed for sex over time for OCC cancer incidence – relating to only marginal increases in female OCC incidence, although incidence rates among males remained at least 1.5 times higher than females across the entire study duration. Increasing OCC cancer among women has previously been reported across the world potentially explained by latent uptake of smoking and alcohol behaviours in women [17, 28, 29].

Low socioeconomic Status (SES) is an established determinant of HNCs [30–33]. A strong inequality gradient was consistently observed across all HNC subsites, including OPC, over the study period. Furthermore, interaction tests showed no temporal interactions with time for HNC incidence. In contrast with previous reports that suggested people with OPC tended to be from higher socioeconomic groups, we found a consistently strong socioeconomic gradient and greater disease burden among those from lower socioeconomic groups [13, 15]. These previous studies evaluating the sociodemographic profile of OPC were smaller clinical cohort or case-control studies mainly conducted in the USA. In these studies, the socioeconomic profile reported may have been skewed towards more affluent participants with access to insurance and healthcare by the nature of recruiting cancer centres. Prior research has shown that high-risk sexual behaviours (which are also associated with an increased risk of HPV infection and HPV-positive OPC), have a similar socioeconomic pattern to the other major HNC risk factors smoking and alcohol consumption – i.e., greater among lower socioeconomic groups [34, 35]. Ultimately, this study contradicts suggestions that the socioeconomic pattern of OPC differs from other HNC subsites and that this pattern is not changing over time. These findings are consistent with previous studies of HNC stage and mortality in Scotland, where similar inequality patterns have been observed [33, 36]. The strong inequality gradient observed in this study highlights the need for targeted primary and secondary prevention strategies to reach at risk groups in socioeconomically deprived areas.

Our study revealed that there been no substantial shift in the age profile of people with HNC. While other reports suggest people with HNC are increasingly of a younger age [13, 28] we found that rates generally increased with age-group and there was no consistent shift to younger age profile over time across all HNC subsites. This was supported by the interaction test results which revealed no temporal change in the age-profile of people with OPC and larynx cancer and marginal increases in the peak age of OCC and overall HNC. These findings could be explained by an aging population observed in Scotland [37].

The incidence trends observed in this study are in accordance with a global trend of increases in HNC driven by OPC, accompanied by declines in smoking-related cancers (larynx) among higher-income nations [4]. Prior epidemiological analyses of UK and international cancer registry data have also shown similar findings of increasing HNC incidence, with rapidly rising OPC rates, declining larynx cancer incidence, and plateauing OCC rates [6, 7, 25, 28, 38]. Our analysis was constrained by a lack of individual patient data but the findings were consistent with behavioural trends in the literature. The stabilisation of OCC rates may be explained by persisting alcohol behaviours and the stronger synergistic effects of alcohol and tobacco behaviours associated with cancers of the oral cavity. Increases in OPC are suggestive of a growing burden of HPV-mediated disease [39–41]. The reduction of larynx cancer rates may be explained by declining tobacco smoking trends [42], while OCC – which is also associated with smoking and the HNC subsite most strongly associated with alcohol drinking [43] – may not be changing due to persisting levels of harmful alcohol consumption [42, 44].

This study had several strengths. Notably, the use of high-quality data recorded and kept by the Scottish cancer registry which allows for a robust, population-wide analysis of HNC trends over time. The registry uses a thorough verification process when recording cases, in addition to quality and accuracy checks, with high levels of accuracy and limited numbers of data discrepancies reported [45, 46]. A robust analysis approach was employed to assess the sociodemographic profile of HNC and subsites including assessing interaction with time.

This analysis had some limitations; the Scottish Cancer registry does not collect tumour HPV data, meaning we were unable to compare the sociodemographics of people with HPV-positive and HPV-negative OPC directly. Future work should investigate the associations between the sociodemographics of people with HPV-positive and HPV-negative OPC in a similarly large study. The registry also does not possess behaviour (smoking / alcohol consumption), nor does the registry include individual-level socioeconomic metrics, e.g. education or income. While postcode-derived (data zone) Indexes of Multiple Deprivation can provide some insight into SES, they are limited by the assumption that individuals within areas are all socioeconomically homogenous. Nevertheless, SIMD is considered a comparable and powerful measure of socioeconomic deprivation at the community level [23, 47].

## Conclusions

We have shown that the sociodemographic profile in terms of age, sex, and socioeconomic background of the incidence burden of head and neck cancers has remained largely unchanged in the last 20 years despite increasing incidence rates which have been driven by rises in oropharynx cancer. These findings are important to help inform efforts to stratify and target prevention, early detection, and cancer services to reach those at the greatest risk. The stark inequality gradient in HNC incidence across all subsites reinforces the importance of upstream tobacco and alcohol regulation, with universal and proportionate prevention strategies to tackle such wide socioeconomic inequalities.

## Supplementary information

Supplementary Figure

## Data Availability

The datasets analysed during the current study are not publicly available due to the nature of Cancer Registry data but are available from Public Health Scotland (PHS) on request and ethical approval.
