# Supplementary material for: Head and neck cancer incidence is rising but the sociodemographic profile is unchanging: a population epidemiological study (2001–2020)
Source: BJC Rep. 2024 Sep 17;2:71. doi: 10.1038/s44276-024-00089-z (PMC11408244; doi:10.1038/s44276-024-00089-z)
Supplement: Supplementary file 1 — Supplementary Figure [file 44276_2024_89_MOESM1_ESM.docx]

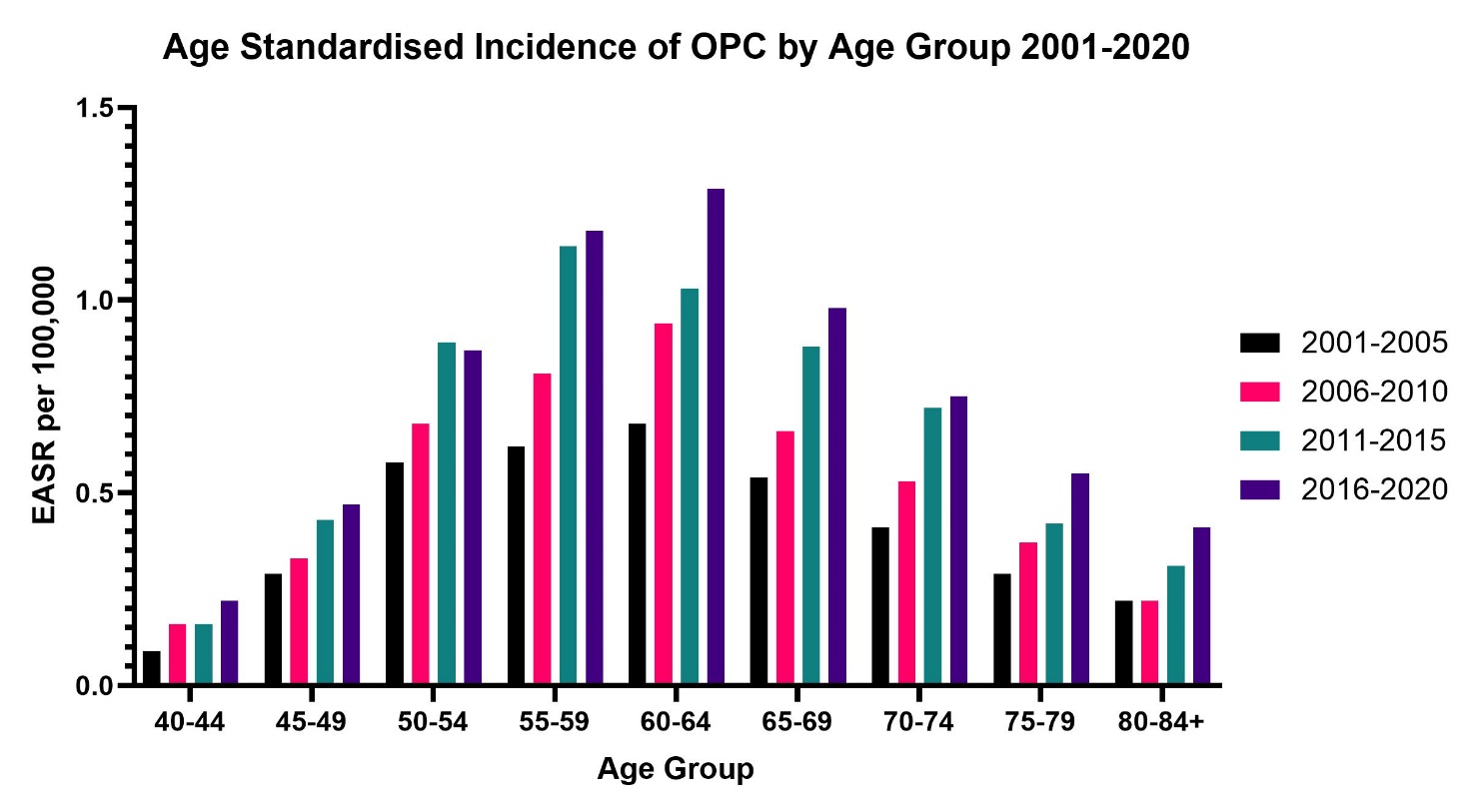


Supplementary Figure 1: Plot of Age-Standardised OPC Incidence Trends by Age-group and 5-year period


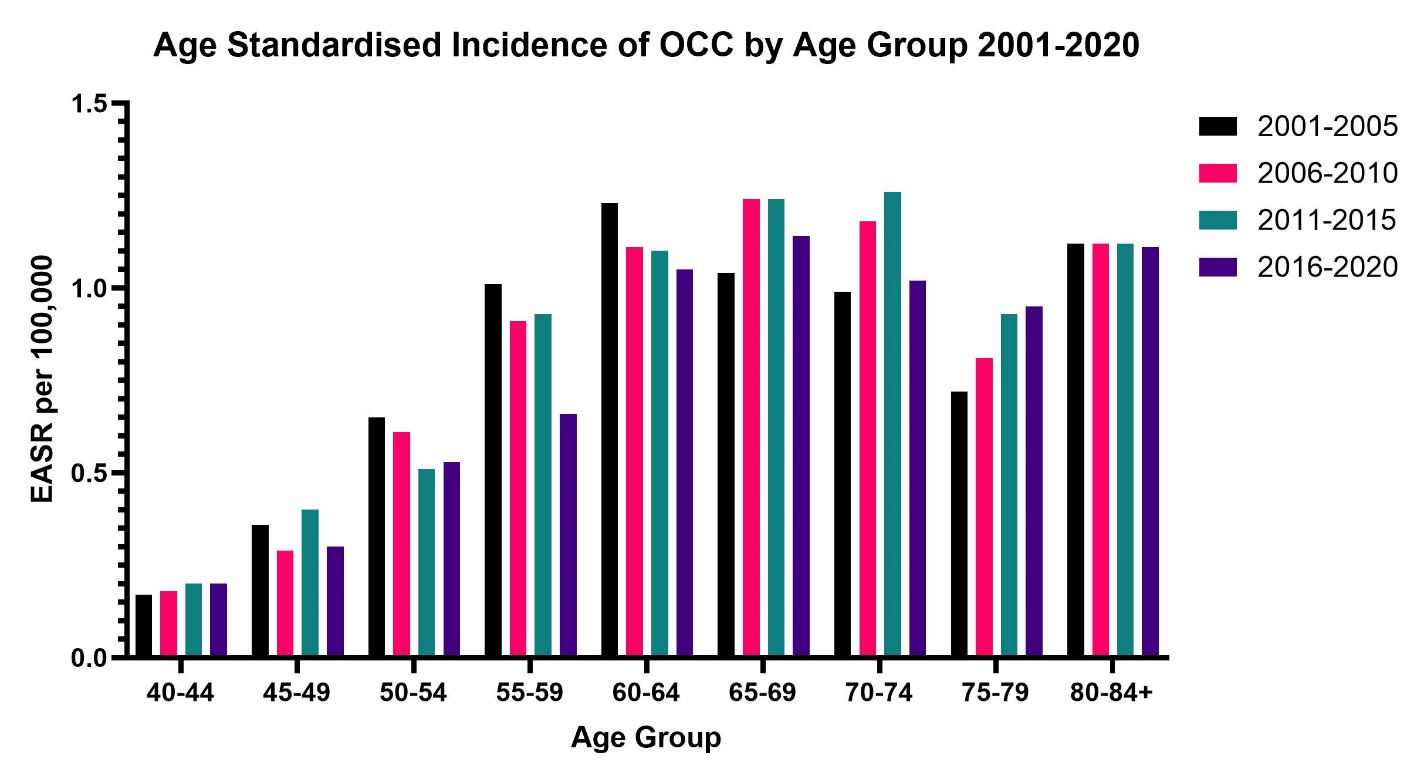


Supplementary Figure 2: Plot of Age-Standardised OCC Incidence Trends by Age-group and 5-year period


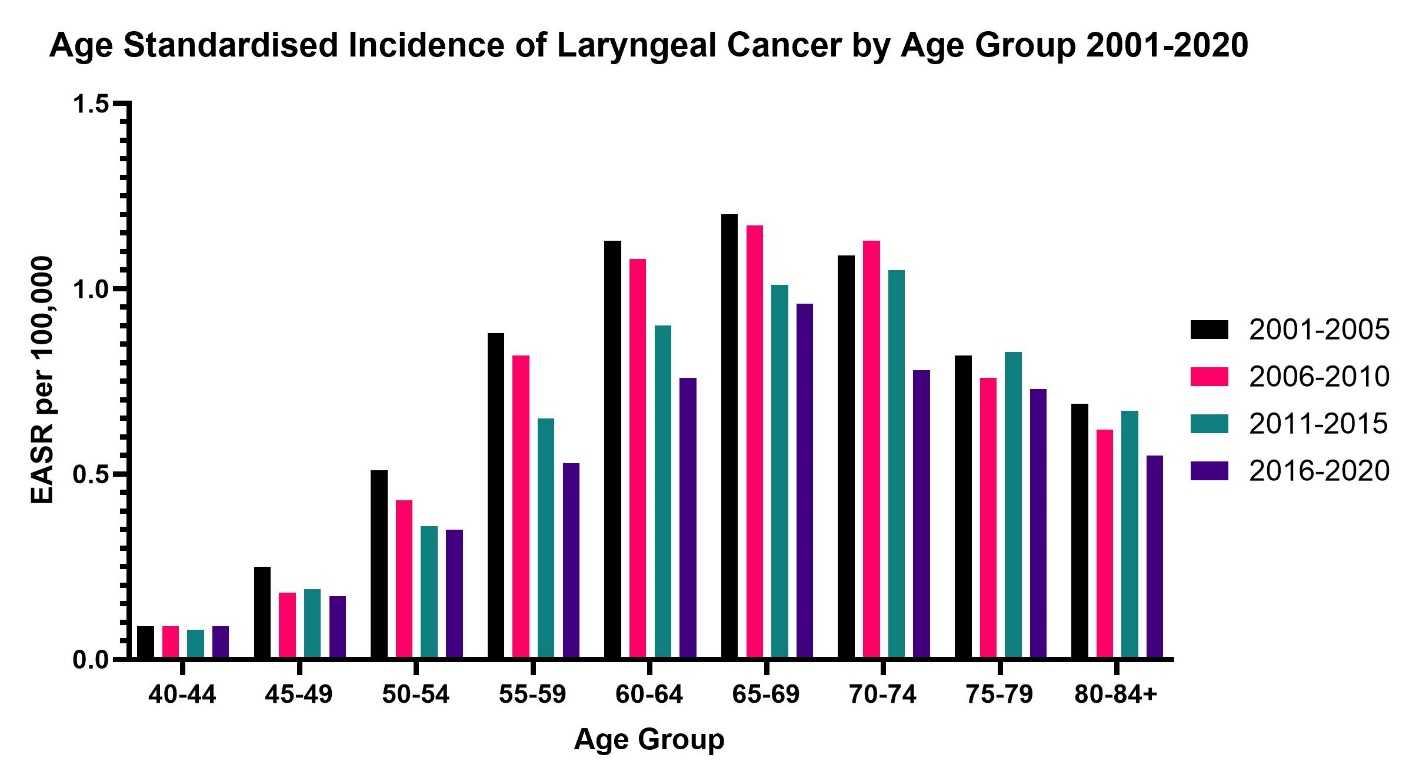


Supplementary Figure 3: Plot of Age-Standardised Laryngeal Cancer Incidence Trends by Age-group and 5-year period


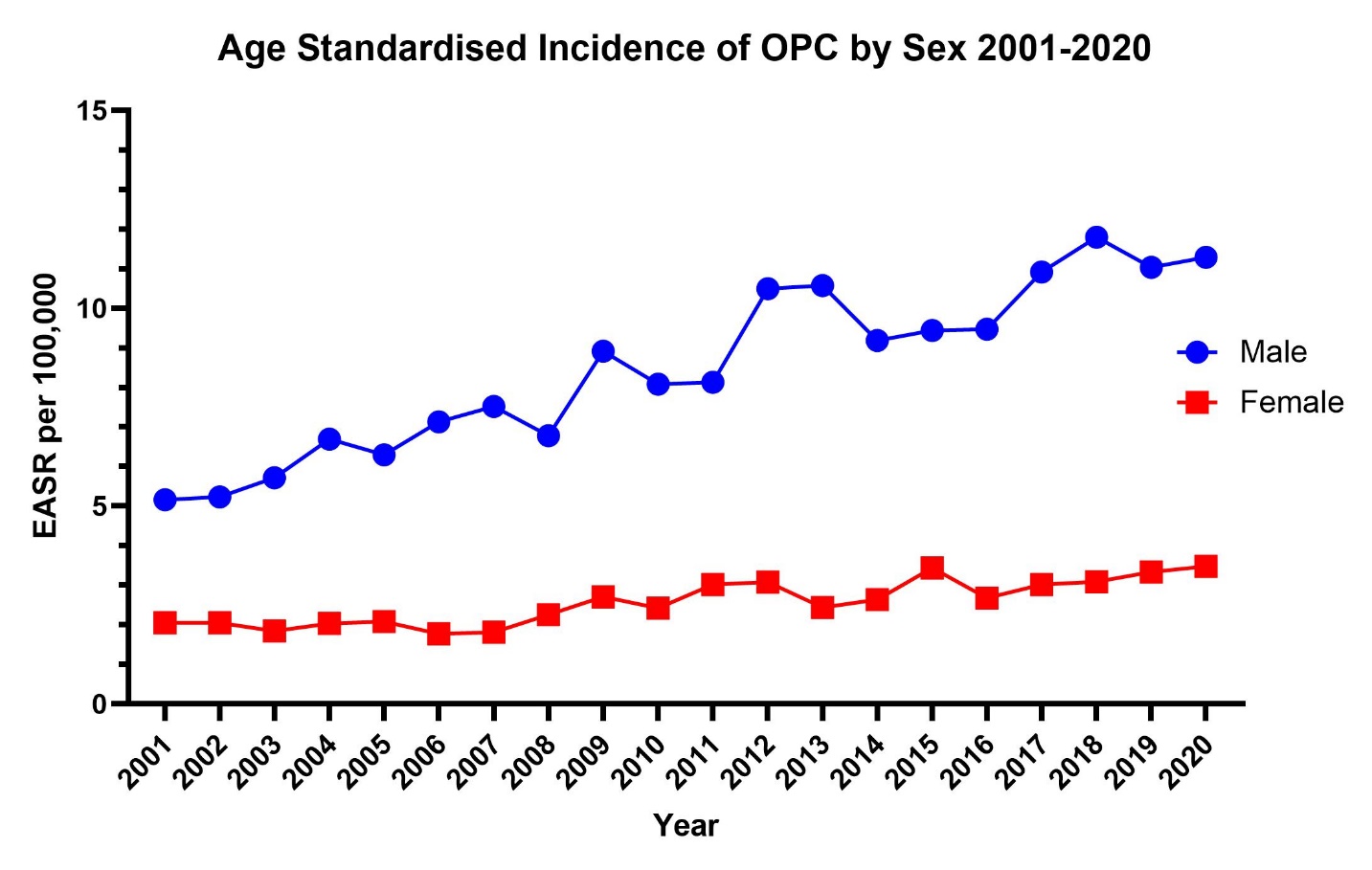


Supplementary Figure 4: Plot of Age-Standardised OPC Incidence Trends by Sex


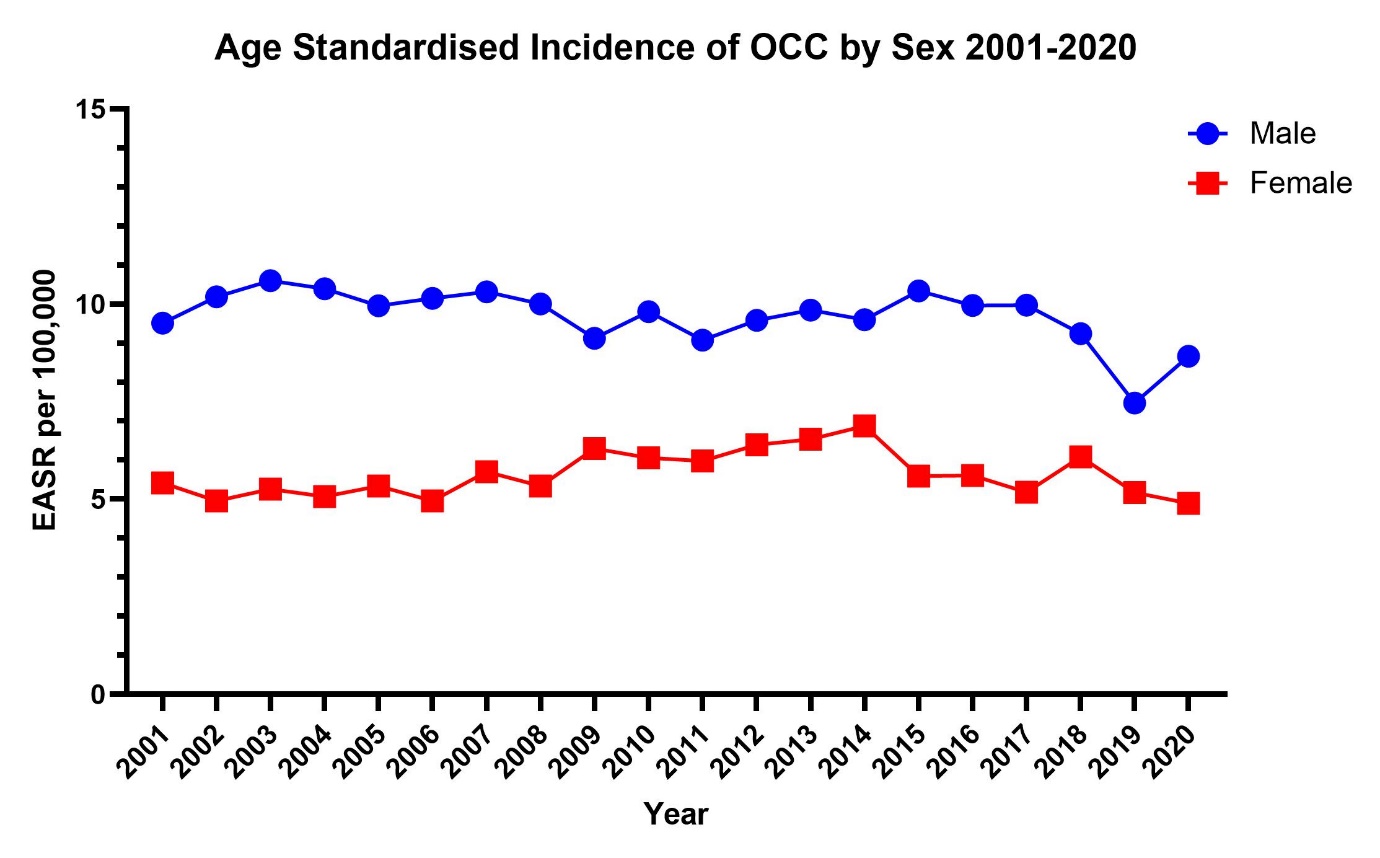


Supplementary Figure 5: Plot of Age-Standardised OCC Incidence Trends by Sex


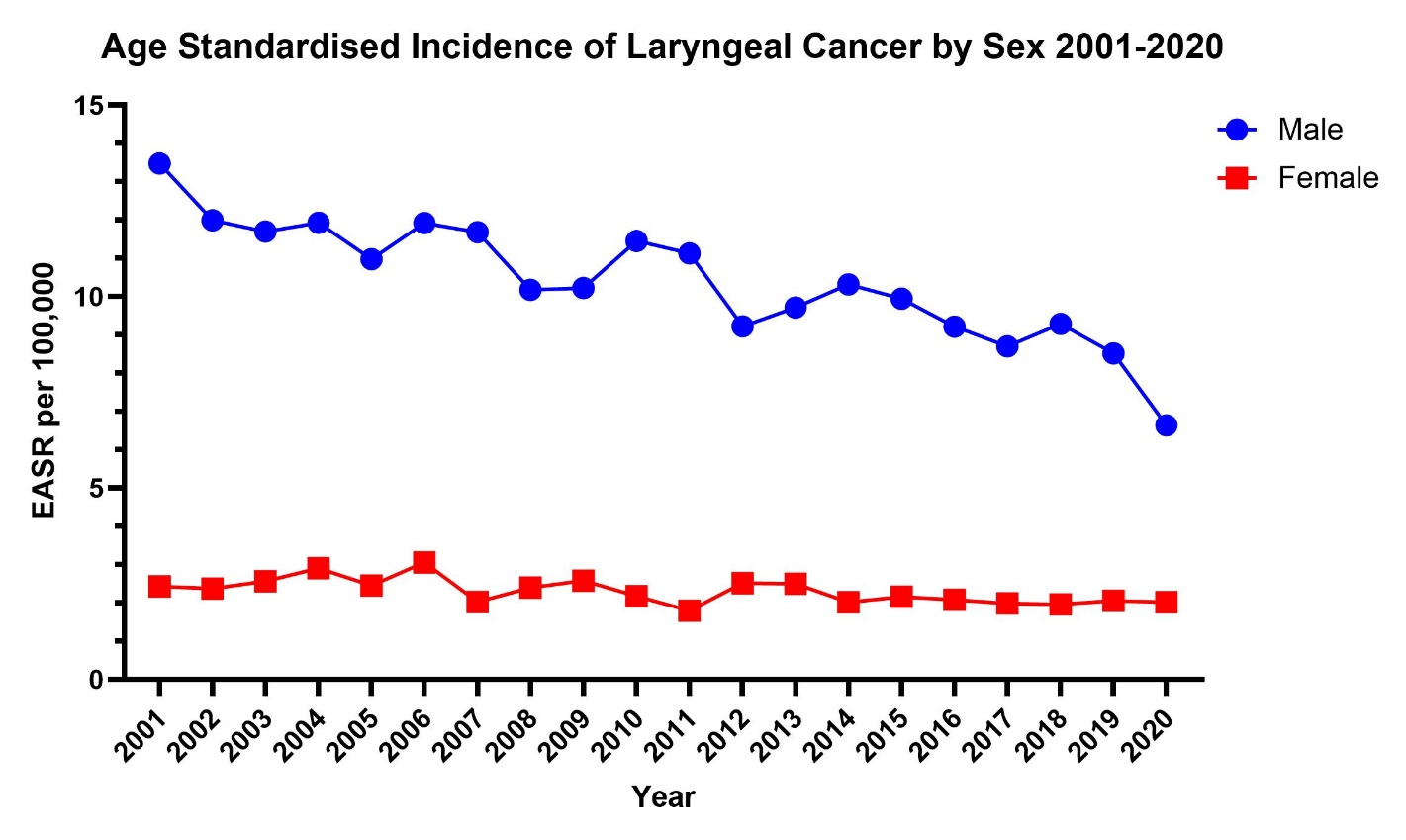


Supplementary Figure 6: Plot of Age-Standardised Laryngeal Cancer Incidence Trends by Sex


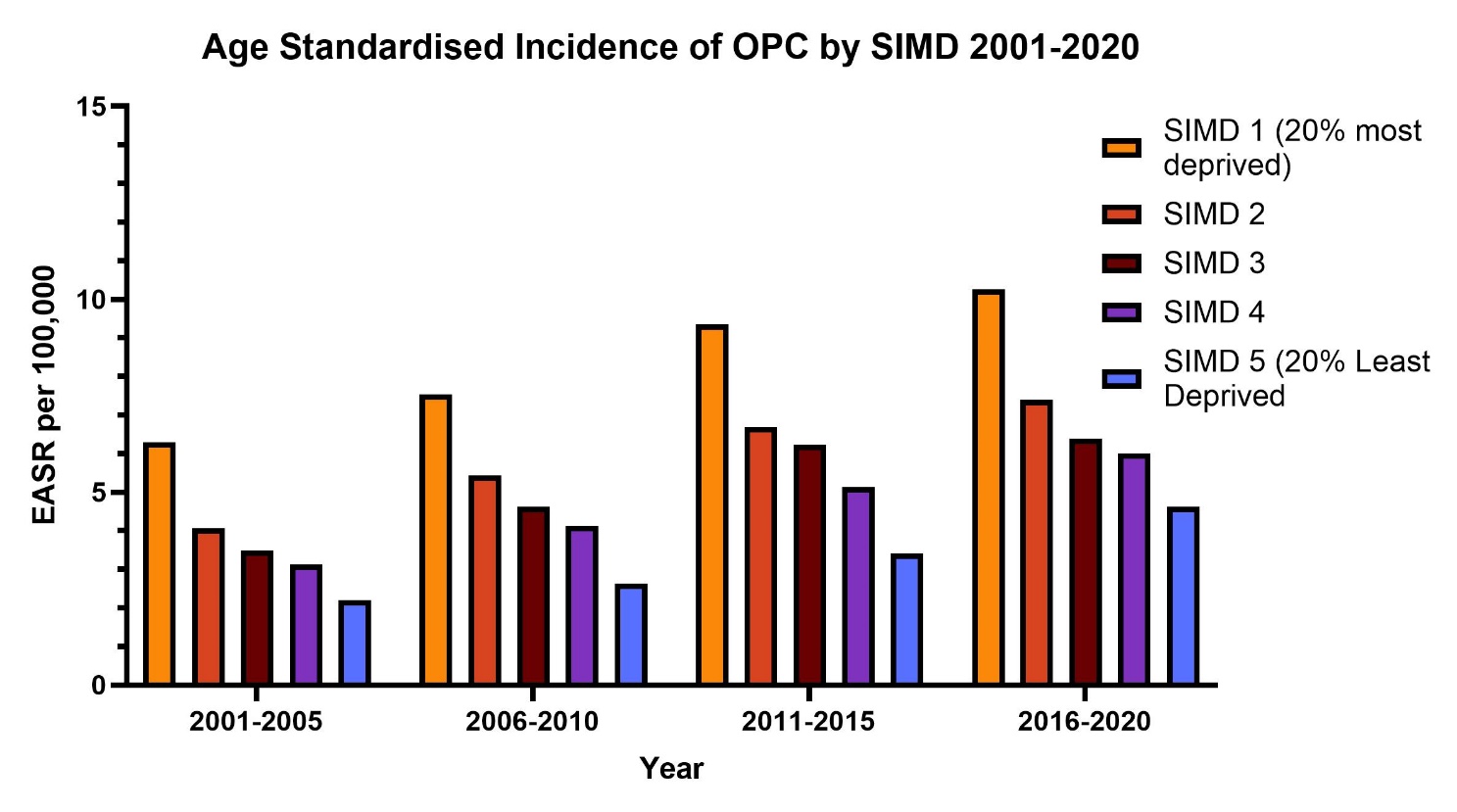


Supplementary Figure 7: Plot of Age-Standardised OPC Incidence Trends by SIMD Quintile and 5-year period


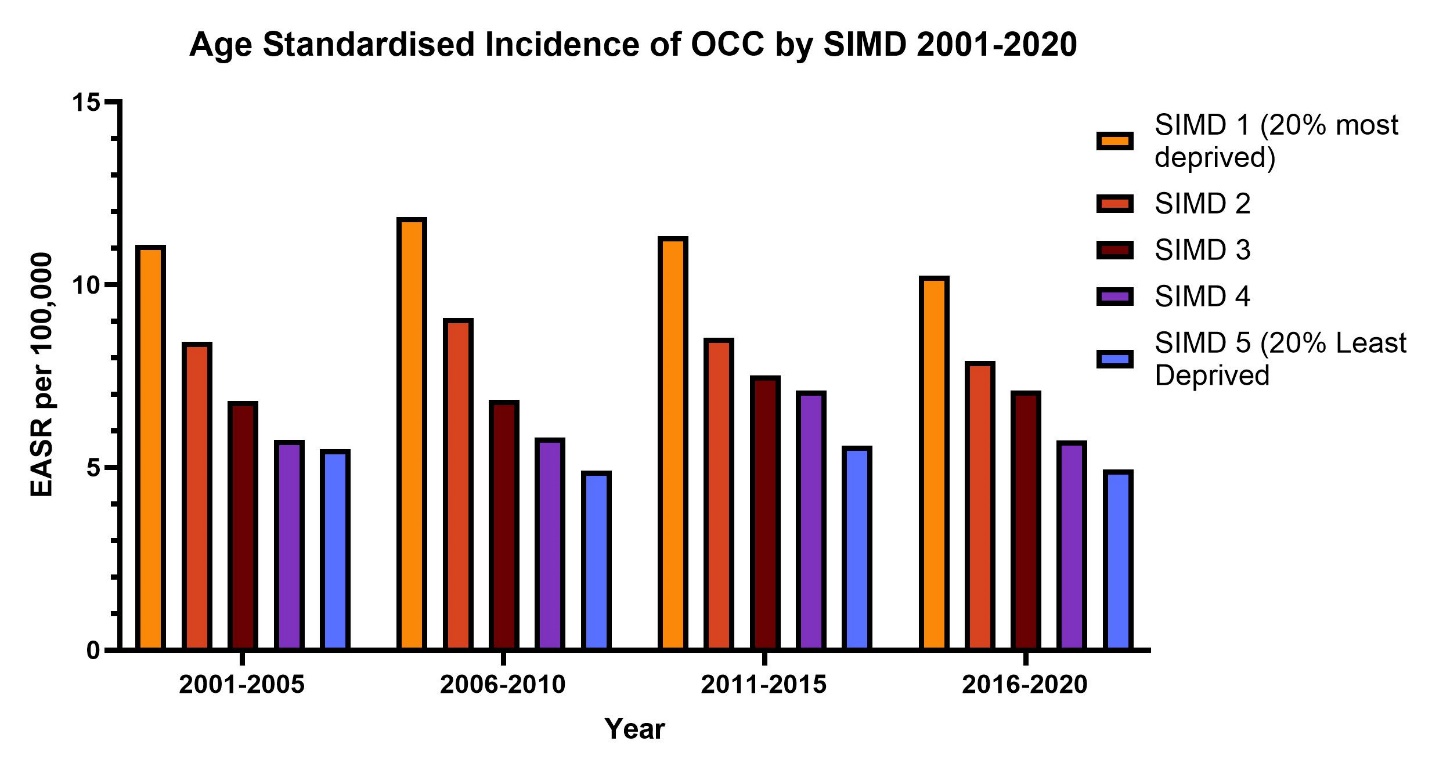


Supplementary Figure 8: Plot of Age-Standardised OCC Incidence Trends by SIMD Quintile and 5-year period


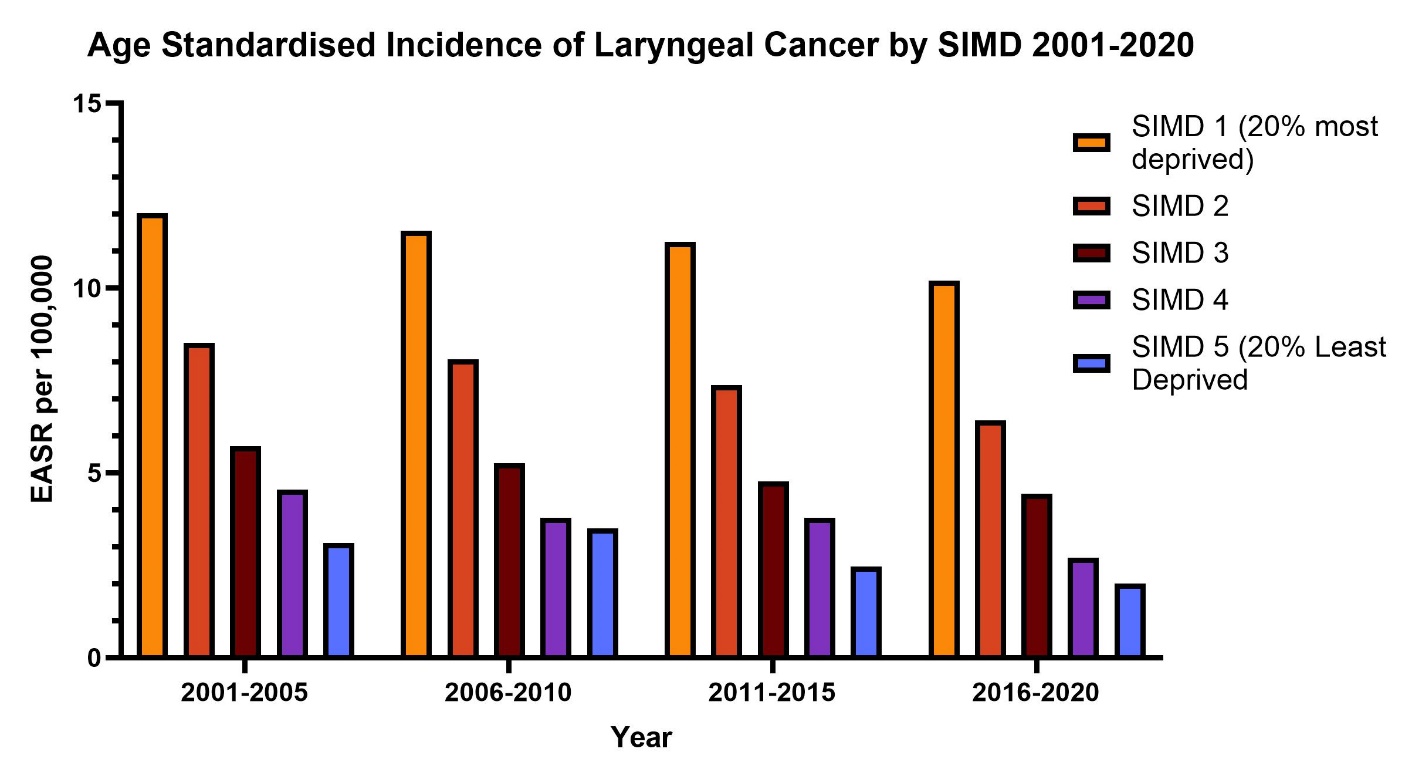


Supplementary Figure 9: Plot of Age-Standardised Laryngeal Cancer Incidence Trends by SIMD Quintile and 5-year period
